# Early Repolarization vs. Acute Pericarditis Morphology: A Case Report of Electrocardiographic Mimicry

**DOI:** 10.7759/cureus.4468

**Published:** 2019-04-16

**Authors:** Everett Rogers, Amanda Maddrey, Christina E Stamoolis, Marc M Kesselman

**Affiliations:** 1 Internal Medicine, Nova Southeastern University Dr. Kiran C. Patel College of Osteopathic Medicine, Davie, USA; 2 Biological Sciences, University of South Florida, St. Petersburg , USA; 3 Family Medicine, St. Petersburg General Hospital, St. Petersburg, USA; 4 Rheumatology, Nova Southeastern University Dr. Kiran C. Patel College of Osteopathic Medicine, Davie, USA

**Keywords:** early repolarization, pericarditis, electrocardiogram, unnecessary tests, unnecessary treatment, electrophysiology

## Abstract

A 27-year-old male presented to the outpatient clinic with a two-week history of daily episodes of palpitations, chest pain, and shortness of breath. He also complained of fatigue and nausea that continued after he recovered from an upper respiratory infection (URI) one month prior. Of note, he described the chest pain as increasing in intensity when sitting or standing upright. Auscultation revealed regular rate and rhythm with no audible rubs or murmurs. An electrocardiogram (ECG) was performed and showed diffuse ST-segment elevations that the machine interpreted as pericarditis. Based on the patient’s symptoms and ECG findings, he was sent for an evaluation by cardiology. After he failed a trial of non-steroidal anti-inflammatory drugs (NSAIDs), the patient was started on colchicine and his symptoms ultimately resolved within a few weeks. Review of his records showed an ECG performed in the emergency department (ED) a year prior demonstrated morphology consistent with early repolarization (ER). The ECG morphology of ER, acute pericarditis (AP), and even acute myocardial infarction (AMI) can often be similar and difficult to differentiate. In this patient, confusing ER with AP may have led to unnecessary evaluation and treatment by a specialist.

## Introduction

After it was first described by Shipley and Hallaran in 1936, early repolarization was long thought to be a benign normal variant of electrocardiogram (ECG) morphology [1]. However, this paradigm has started to shift in the past decade after several studies linked ER to fatal ventricular arrhythmias. This includes a 2008 study by Haïssaguerre et al. that found approximately one-third of case subjects were discovered to have had early repolarization (ER) following cardiac arrest due to idiopathic ventricular fibrillation [2]. Some studies have even posited that ER is an independent predictor of ventricular fibrillation after acute myocardial infarction (AMI) [3]. Additionally, ER morphology can often be difficult to distinguish from acute pericarditis (AP) or AMI on an ECG tracing, especially in certain clinical settings, as highlighted by Turnipseed et al. [4]. This case presents a young man with ECG changes and symptoms suggestive of AP, but who likely only had persistent ER morphology and a lingering upper respiratory infection (URI).

## Case presentation

**Figure 1:**
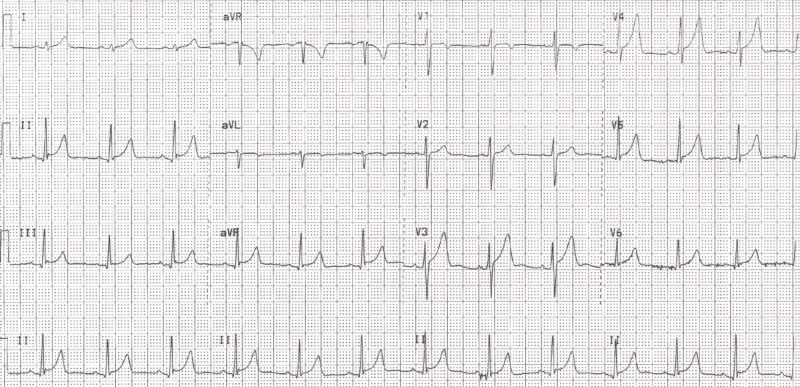
ECG from initial outpatient clinic visit showing extensive ST elevations, suggestive of possible pericarditis ECG, electrocardiogram

**Figure 2:**
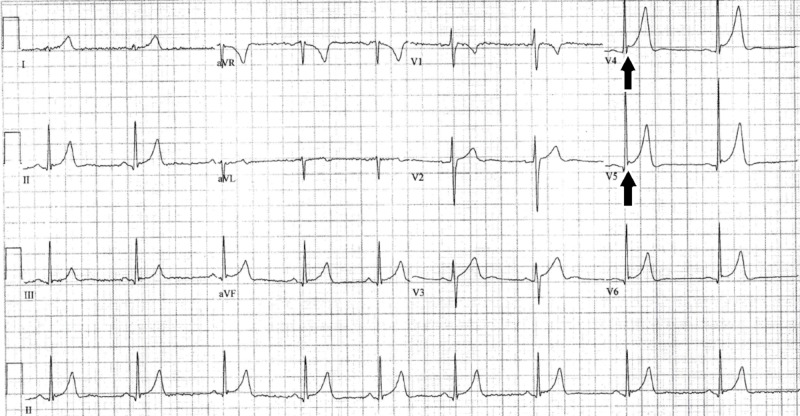
ECG showing bradycardia with ER morphology from ED visit for abdominal pain one year prior to initial outpatient presentation Black arrows indicate J-point notching and elevation typical of early repolarization, especially in the precordial leads. ECG, electrocardiogram; ER, early repolarization

A 27-year-old male presented to the outpatient clinic with two weeks of lightheadedness without syncope, occasional shortness of breath, and four episodes of palpitations per day that had progressed to chest pain which worsened in an upright position. He also experienced fatigue, hot flashes, and occasional nausea for the past month following a URI. Past medical history was significant for pulmonary nodules found to be stable on serial computerized tomography scans. He denied tobacco or alcohol use, but admitted to marijuana use that ceased when the nodules were discovered. He was thin, but athletic, with a body-mass index of 19. He had clear lungs bilaterally, a blood pressure of 115/74 mmHg, a pulse of 70 beats per minute (bpm), and a regular rate and rhythm without rubs or murmurs upon auscultation. An ECG revealed extensive ST-segment elevations suggestive of pericarditis, which was noted by the ECG machine (Figure 1). Review of his medical records revealed an ECG from an ED visit one year prior showing only ER morphology (Figure 2).

The ECG tracing shown in Figure 1 exhibited sinus rhythm at a rate of 74 bpm. ST elevations were present in leads II, III, and aVF, with slightly more pronounced elevations in precordial leads V3 through V5. J-point notching was also evident in the inferior leads. Minimal PR depression was seen in the inferior leads. The ST-segment elevation to T-wave height ratio was less than 0.25 in leads V4 through V6.

Based on his symptoms, history of a recent URI, and the presence of diffuse ST elevations on ECG, the patient was diagnosed with AP. He was prescribed NSAIDs and referred to cardiology for follow-up. The following day he went to the ED for continued chest pain. In the ED, an ECG was done and showed evidence of ER changes (i.e. ST elevations and J-point notching), but was otherwise unremarkable. A chest X-ray revealed no acute changes and, a C-reactive protein level was within normal limits. On follow-up, cardiology diagnosed him with AP. A 24-hour Holter monitoring and an ECG were performed, showing no effusion or other abnormalities. After discussing the risks and benefits of treatment, the patient was started on colchicine. His symptoms ultimately resolved within a few weeks.

## Discussion

The definition of ER is often heterogeneous and commonly varies depending on the publication or authority [5]. This can influence estimates of ER prevalence, which is typically put between 1% and 25% of the general population, with 10% being a reasonable approximation used by the American Heart Association [5-6]. This relatively high percentage of people with the ER variant, combined with its ability to mimic ECG findings in AP and AMI, can lead to significant clinical challenges, particularly for non-cardiologists. In fact, a 2006 study by Turnipseed et al. found that when provided with only the patient’s age, gender, and race, in addition to a corresponding ECG tracing, emergency room physicians misdiagnosed AMI as ER 10% of the time, and misdiagnosed ER as AMI 28% of the time [4]. This study was limited in size, yet it underscores the difficulties in correctly diagnosing ER with even a well-trained eye.

Although the differences can be subtle, there are several distinguishing features that can help differentiate ER from AP. While they both typically feature diffuse, concave ST-segment elevation, PR-segment depression is typically only seen in the latter [7]. While the J-wave is classically notched in multiple leads in ER, it can also be hidden within the terminal portion of the QRS complex, leading to a slurred, rather than notched morphology [8]. Perhaps the most telling, but least immediately appreciable feature differentiating the two is the ST-segment height to T-wave height ratio [7]. This ratio is greater than 0.25 in AP and less than 0.25 in ER, particularly in leads I, V4, V5, and V6 [7]. Interestingly, as seen in Figure 1, this ratio is less than 0.25 in leads V4 through V6, putting this finding at odds with the rest of the ECG signs that pointed towards a diagnosis of AP at the time of this patient's initial presentation. This ratio was less than 0.25 in the ECG done a year prior where the ER morphology was much more appreciable. It is also important to note that the slurring or notching of the terminal QRS complex is often more obvious if the patient is bradycardic - another feature commonly associated with ER [8]. This bradycardia-associated ER morphology accentuation is perhaps why the ECG machine was able to distinguish the pattern in this patient a year earlier, but not in the ED the day following his initial outpatient clinic visit.

ST elevations in a patient with chest pain can make distinguishing ER from AMI difficult. This is due to the fact that chest pain and other clinical symptoms are the most obvious way to distinguish between the two. ST-segment elevation is characteristic of both morphologies and can be a common source of confusion. However, the morphology of these elevations will typically become more convex, rather than concave, as AMI progresses [9].

In patients with ER variants and chest pain due to causes other than infarction of myocardial tissue, the risk of inadvertent administration of fibrinolytics or percutaneous intervention is a significant concern. In fact, a study by Alimurung et al. found that in a group of 264 patients undergoing diagnostic cardiac catheterization, 16 were found to have ER [10]. Of these, exercise testing caused the ST elevations of 13 to return to iso-electric baseline, and the coronary angiograms of 14 were found to be normal [8,10].

Although the arrhythmogenicity of certain ER electrocardiographic phenotypes has been well established, the current literature still lacks homogeneity in the evaluation, and even the definition of ER [2,6]. Although a patient presenting with chest pain requires further evaluation, it is important to maintain a high index of suspicion for ER in order to prevent the unnecessary administration of fibrinolytics or percutaneous intervention, both of which would confer an unfavorable risk to benefit ratio upon the patient [2,8].

## Conclusions

ER is a relatively common finding on ECG. Despite its prevalence, it can be difficult to differentiate from other pathologies, such as AP, in certain clinical settings. ECG machine algorithms are not infallible and can lead physicians astray, as was the case with this patient. When trying to differentiate AP from ER, practitioners should look for the presence of J-point notching, as well as ST-segment to T-wave height ratio to help discern the difference between the two. When working up AP, practitioners should keep ER on their list of differential diagnoses so as not to expose patients to potentially harmful and unnecessary treatment.
